# Sequencing and phylogenetic analysis of the complete chloroplast genome of *Cassia tora* Linn

**DOI:** 10.1080/23802359.2019.1688708

**Published:** 2019-11-13

**Authors:** Binxin Xie, Yanan Gai, Ziyan Zhu, Mingzhi Li, Yucheng Zhao

**Affiliations:** a Department of Resources Science of Traditional Chinese Medicines and State Key Laboratory of Natural Medicines, School of Traditional Chinese Pharmacy, China Pharmaceutical University, Nanjing, Jiangsu, P. R. China;; b Institute of Botany, Jiangsu Province and Chinese Academy of Sciences, Nanjing, Jiangsu, P. R. China;; c Biodata Biotechnology Co. Ltd., Hefei, Anhui, China

**Keywords:** Complete chloroplast genome, phylogenetic analysis, *Cassia tora*

## Abstract

*Cassia tora* Linn. is widely distributed in South-East Asia and South-West Pacific as an important weed. It has many pharmacological activities including anti-allergic, anti-hepatotoxic, and remedy in skin diseases. In this study, we assembled and characterized the complete chloroplast genome sequence of *C. tora* from high-throughput sequencing data. The chloroplast genome was 162,426 bp in length, consisting of large single-copy (LSC) and small single-copy (SSC) regions of 90,843 bp and 18,001 bp, respectively, which were separated by a pair of 26,791 bp inverted repeat (IR) regions. The genome is predicted to contain 131 genes, including 84 protein-coding genes, 37 tRNA genes, and 8 rRNA genes. The overall GC content of the genome is 36.0%. A phylogenetic tree reconstructed by 32 chloroplast genomes reveals that *C. tora* is mostly related to *Senna occidentalis*. The work reported the firstly complete chloroplast genome of *C. tora* which may provide useful information to the evolution of Cassieae Bronn.

*Cassia tora* is a medicinal plant that is widely distributed in South-East Asia and South-West Pacific as an important weed. The leaf of *C. tora* has been used to manage haemorrhoids, skin infections, cough, pneumonia, stomach ache, ulcer, and fever for many years (Adamu et al. 2005). Pharmacological analysis indicated that it has the ability to anti-hepatotoxic, anti-allergic, anti-mutagenic, radical scavenging, hypoglycaemic, and anti-microbialactivities (Rejiya et al. 2009; Chethana et al. 2017). Alkaloids, phenols, anthraquinones, glycosides, flavonoids, and saponins are the major secondary metabolites have been reported in *C. tora* (Yen et al. 1998; Aldouri 2000). However, little is known in its biosynthesis mechanism. In addition, the phylogenetic position of *C. tora* and the genus Cassieae Bronn is still unresolved. In this study, we first reported the complete chloroplast (cp) genome of *C. tora*, and its phylogenetic analysis is also investigated which provides informatics data for the phylogeny of genus Cassieae Bronn.

The fresh leaves of *C. tora* from Fuyang, Anhui, China (32°35′N, 114°50′E) were used for genomic DNA extraction. Specimens were stored in the Department of Resources Science of Traditional Chinese Medicines of China pharmaceutical University with the accession number of JMZ20190715XBX-4. Total genomic DNA was extracted with a FastPure Plant DNA Isolation Mini Kit (Vazyme, Nanjing, China). The whole-genome sequencing was conducted by Hefei Biodata Biotechnologies Inc. (Hefei, China) on the Illumina Hiseq platform. The filtered sequences were assembled using the program SPAdes assembler 3.10.0 (Anton et al. 2012). Annotation was performed using the DOGMA and BLAST searches (Wyman et al. 2004). The cp genome of *C. tora* was determined to comprise a 162,426 bp double-stranded, circular DNA (GenBank accession no. MN480300), which containing two inverted repeat (IR) regions of 26,791 bp, separated by large single-copy (LSC) and small single-copy (SSC) regions of 90,843 bp and 18,001 bp, respectively. The overall GC content of *C. tora* cp genome is 36.0% and the corresponding values in LSC, SSC, and IR regions are 33.3, 30.2, and 42.3%, respectively. The cp genome was predicted to contain 131 genes, including 84 protein-coding genes, 37 tRNA genes, and 8 rRNA genes. Eleven genes contained two exons and four genes (ycf3, clpP, and two rps12) contained thee exons.

To investigate its taxonomic status, Alignment was performed on the 12 cp genome sequences using MAFFT v7.307, and a maximum likelihood (ML) tree was constructed by FastTree version 2.1.10 (Price et al. 2010; Kazutaka and Standley 2013). As expected, *Senna ossidentalis* is the most related species to *C. tora*, with bootstrap support values of 100% (Figure 1). The complete cp genome sequence of *C. tora* will provide a useful resource for the conservation genetics of this species as well as for the phylogenetic studies of Cassieae Bronn.

**Figure 1.**
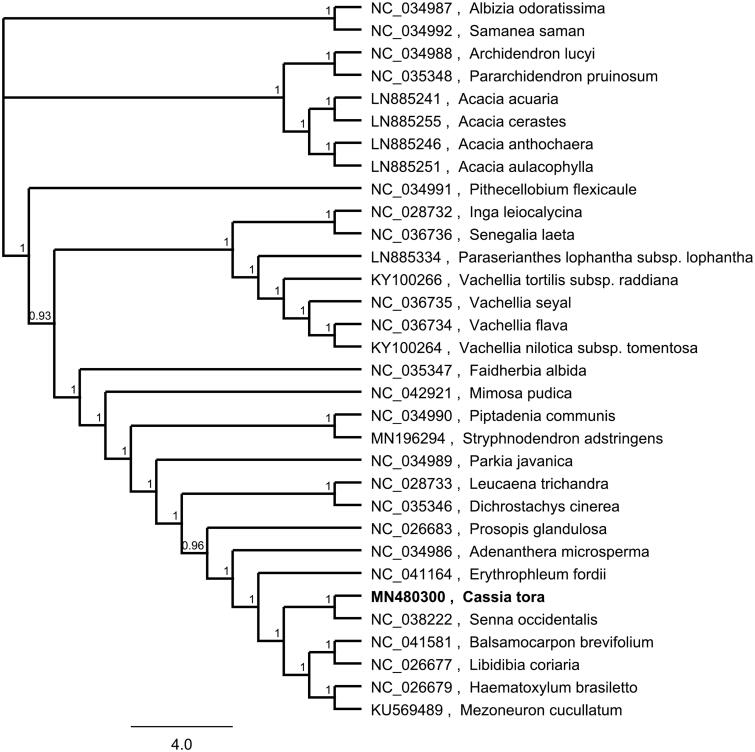
Phylogenetic tree inferred by maximum likelihood (ML) method based on 32 representative species. A total of 1000 bootstrap replicates were computed and the bootstrap support values are shown at the branches. GenBank accession numbers were shown in Figure 1.
